# Sarcoidosis: experience in a Mexican ophthalmological clinic

**DOI:** 10.1186/s12886-023-03081-2

**Published:** 2023-07-20

**Authors:** Javier Alejandro Salazar-Rodríguez, Diana Sarmiento-Forero, Claudia Hubbe-Tena, Luz-Elena Concha-del-Rio

**Affiliations:** 1 Asociacion Para Evitar la Ceguera en México Hospital Dr. Luis Sánchez Bulnes, Mexico City, Mexico; 2 Inflammatory Eye Disease Clinic, Asociacion para Evitar la Ceguera en México, I. A. P, México City, Mexico

**Keywords:** Ophthalmology, Sarcoidosis, Uveitis, México

## Abstract

**Background:**

Sarcoidosis is an inflammatory disorder in which patients frequently develop ocular manifestations that precede systemic involvement, sometimes it even presents as an ocular isolated form of the disease. The purpose of this study is to report the ocular and systemic manifestations of sarcoidosis in a series of Mexican patients, as there is a low incidence of the disease in this population.

**Methods:**

A retrospective case series of patients with positive classification criteria for sarcoidosis who attended Asociacion Para Evitar la Ceguera en Mexico, IAP between 2011 and 2022. Descriptive statistics were used to report the clinical, laboratory, and imaging findings and treatment. Numerical results were presented using median values and first and third quartiles for distribution.

**Results:**

Fourteen patients were included in this study, 10 of them had definite ocular sarcoidosis (biopsy-proven), 4 had presumed ocular sarcoidosis. The median age of onset was 52 (34; 67), with a predominance of female patients (71.4%). Ten patients (71.4%) debuted with ocular manifestations. The most common forms of ocular involvement were bilateral anterior uveitis (50%) and panuveitis (28.6%). Median follow-up was 24 (13–49) months.

**Conclusions:**

Sarcoidosis is a rare, underdiagnosed condition in Mexico and ocular involvement can be an early manifestation of the disease. Ophthalmologists should be alert to the signs of ocular sarcoidosis and collaborate with a multidisciplinary team to screen for systemic involvement if suspicion is high.

## Background

Sarcoidosis is an inflammatory disorder of unknown cause, characterized by systemic inflammation and the formation of noncaseating epithelioid cell granulomas, which can affect practically every organ [1]. It’s incidence is estimated to be 5.9/100,000 for men and 6.3/100,000 for women in United States, [2] and it is a relatively common cause of uveitis in European patients [3].

Despite this, sarcoidosis in Latin America is rare, and the information on its prevalence, risk, and morbidity in Hispanic patients is limited [4–7]. There are only 28 published cases of sarcoidosis in Mexico and only 5 which report ocular manifestations (Table 1) [4, 8–14].

**Table 1:** Sarcoidosis in Mexico 1991–2022

Study	Year	n	Definite with biopsy (WASOG)	Definite Ocular sarcoidosis (IWOS)
Arévalo-López (8)	1991	1	0	0
Estrada-Correa (9)	2006	1	1	0
Fernandez-Sanchez (10)	2012	1	1	0
Carrillo-Pérez (4)	2015	21	21	4 (uveitis)
Peralta-Gómez (11)	2018	1	1	1 (dacryocystitis)
Vega-Cornejo (12)	2019	1	1	0
Almaguer-Morales (13)	2020	1	1	0
Solis (14)	2020	1	1	0
Salazar-Rodríguez	2022	14	10	10
Total		42	37	14

N (number of patients) WASOG (World Association of Sarcoidosis and Other Granulomatous Disorders) [1] IWOS (International Criteria for the Diagnosis of Ocular Sarcoidosis) [21]

There is a wide spectrum of systemic manifestations of sarcoidosis that encompass many specialties in medicine. Lungs are the most affected organ in the disease (more than 90% of all cases) [15]. Although these manifestations are mainly non-specific of sarcoidosis, such as dyspnea, cough, and chest pain, lung imaging studies help identify specific signs that support the diagnosis. The two main imaging findings in pulmonary sarcoidosis are bilateral hilar lymphadenopathy (BHL) and perilymphatic pulmonary nodules [16].

The second most affected organ in patients with sarcoidosis is the eye. Up to 30–50% of patients with systemic sarcoidosis will show eye involvement, or it can only be confined to the eye. The two main ocular manifestations are keratoconjunctivitis sicca and uveitis. Regarding ocular inflammation, anterior uveitis and panuveitis are the most common form of presentation, although the disease can affect any section of the eye [17–20].

Ocular manifestations are often bilateral. These include orbital and adnexal non-specific manifestations, such as keratoconjunctivitis sicca (dry eye), dacryocystitis, conjunctival granulomas, and less commonly diffuse orbital inflammation, ocular nerve palsy, and scleral granulomas [22].

Specific ocular manifestations support clinical diagnosis and help differentiate sarcoid uveitis from other types of inflammation. These are often accompanied by a sudden onset of pain or loss of visual acuity and include anterior segment signs such as mutton fat keratic precipitates (granulomatous uveitis) iris nodules either at the pupillary margin (Koeppe) or in the stroma (Busacca), Berlin nodules (trabecular meshwork nodules) and anterior tent-shaped synechiae at the iridocorneal angle. There are also intermediate and posterior inflammatory manifestations like snowbanking and snowball vitreous opacities, candle wax vascular shedding (retinal periphlebitis), microaneurysms, and optic disc nodules (granulomas). These manifestations guide the diagnosis of ocular sarcoidosis [1, 19, 21, 23].

Other manifestations include specific syndromes that have ocular and systemic manifestations, which help diagnose sarcoidosis without the need for a confirmatory biopsy, and include Heerfordt–Waldenström syndrome (uveoparotid fever), a combination of facial palsy, parotid gland enlargement, uveitis, and low/grade fever, and Löfgren syndrome, a combination of fever, BHL, erythema nodosum with migratory arthritis including or without ankle [22].

The aim of this study is to report the ocular manifestations, systemic involvement, management, and outcome of sarcoidosis in a Mexican ophthalmology clinic.

## Methods

Medical charts, and records from patients who arrived at the Inflammatory Eye Disease Clinic from 2011 to 2022, in Association Para Evitar la Ceguera en México were retrieved and classified using the revised International Criteria for the Diagnosis of Ocular Sarcoidosis (IWOS) and the Standardization of Uveitis Nomenclature (SUN) classification criteria for ocular sarcoidosis [2, 21, 24]. Patients classified as “presumed sarcoidosis” and “definite sarcoidosis” were included. Initial and final Snellen best-corrected visual acuity (BCVA) were documented.

Furthermore, the clinical evolution of each patient was classified using the SUN nomenclature of clinical status which includes inactive (grade 0 cells on anterior chamber), worsening activity (two-step increase in inflammation of anterior chamber cells or vitreous haze, or increase from 3 + to 4+), improved activity (two-step decrease in the level of inflammation of anterior chamber cells or vitreous haze, or decrease to grade 0), remission (inactive disease for > 3 months after discontinuation of treatment) [24].

A rheumatologist performed a systemic evaluation with laboratory and imaging studies. Systemic involvement findings were collected using the updated World Association of Sarcoidosis and Other Granulomatous Disorders (WASOG) tool [1]. Diagnosis was based on the guidelines of the American [25] and British [26] Thoracic Societies. Classic syndromes such as Löfgren (a triad of fever, erythema nodosum, bilateral hilar lymphadenopathy, and associated migratory arthritis, including ankle) and Heerfordt-Waldenström (parotid gland enlargement, facial palsy, anterior uveitis, and fever) were also included.

Laboratory studies included angiotensin-converting enzyme (ACE), which is an enzyme produced by epithelioid cells derived from activated macrophages (characteristic of sarcoidosis) and lysozyme (LYS) is an enzyme produced by giant epithelioid cells, that reflects the presence of granulomas. Both were considered high when they exceeded > 120% of the laboratory reference value, blood serum calcium, and lymphocyte count values were collected retrospectively when available. Hypercalcemia was defined as serum calcium > 10.2 mequ/L and lymphopenia as < 1000 lymphocytes. Also, infectious diseases were ruled out, including syphilis, with a negative venereal disease research laboratory (VDRL) test and FTA-ABS, and tuberculosis with the tuberculin test (PPD) and/or IGRA test [27]. If a patient presented with a positive PPD test for tuberculosis, a negative IGRA test and a positive biopsy for sarcoidosis could still discard tuberculosis [2].

Pulmonary involvement was defined as the presence of micronodules, atelectasis, hilar lymphadenopathies, or parenchymal changes without predisposing factors. The amount of involvement was categorized using the Scadding test for chest X-rays [16]. Chest computed tomography (CT) was also included when available [28].

Confirmation was achieved with the presence of non-caseating granulomas, Schawman, or asteroid bodies in a tissue biopsy or a classic specific form of sarcoidosis, such as Heerfordt´s or Lofgren´s syndromes [2, 21].

Results were analyzed using the IBM Statistical Package for the Social Sciences (SPSS) 25.0. Descriptive statistics were used to report the clinical, laboratory, and imaging data. Numerical results were presented using median values and first and third quartiles for distribution.

The study was approved by the Institutional Review Board (IRB) of our hospital and adhered to the tenets of the Declaration of Helsinki.

## Results

Fourteen patients with a clinical and/or histological diagnosis of sarcoidosis were identified. The median age was 52 years (27; 67), and 71.4% (n = 10) were female. The median follow-up was 24 (13–49) months. Two patients had tattoos and one had a history of smoking. Ten (71.4%) patients were defined as a biopsy-proven definite diagnosis of ocular sarcoidosis (Table 2), and 4 (28.6%) as a presumed diagnosis by bilateral hilar lymphadenopathy (BHL) and classical ocular signs.

**Table 2:** Characteristics of patients with definite ocular sarcoidosis

	Age	Sex	Ocular	Systemic	Debut	Tests and imaging	Biopsy
1	53	F	Bilateral anterior uveitis (granulomatous) + Dacryoadenitis	Skin granuloma	Ocular	ACE (+) LYS (+) SCAD (3) CT (BHL)	Lachrymal gland
2	70	F	Bilateral anterior uveitis (granulomatous)	Erythema nodosum	Ocular	ACE (-) SCAD (0) CT (pneumonitis)	Skin
3	28	M	Bilateral anterior uveitis (granulomatous)	Löfgren syndrome	Ocular	ACE (+) LYS (+) CT (BHL)	Skin
4	70	F	Bilateral anterior uveitis (non-granulomatous)	Dyspnea, skin plaques, lymphadenopathy	Ocular	ACE (-) LYS (+) PPD (+) SCAD (3) CT (pneumonitis)	Skin
5	56	F	Bilateral panuveitis	Dyspnea	Ocular	ACE (+) LYS (+) SCAD (3) CT (nodules)	Bronchi
6	66	F	Bilateral panuveitis	Arthritis	Ocular	ACE (+) LYS (-) CT (nodules)	Vitreous
7	42	F	Bilateral intermediate uveitis	Heerfordt syndrome	Pulmonary (dyspnea)	SCAD (2) CT (BHL)	Bronchi
8	21	M	Bilateral anterior uveitis (non-granulomatous)	Skin granuloma	Skin	CT (BHL)	Skin
9	24	M	Bilateral anterior uveitis (granulomatous)	Skin granuloma	Skin	-	Skin
10	68	F	Bilateral anterior uveitis (non-granulomatous) + dacryoadenitis	Cough, dyspnea, fever, weight loss	Pulmonary	SCAD (3) CT (BHL)	Lachrymal gland

ACE (angiotensin converting enzyme), ADA (adalimumab), AZA (azathioprine), BHL (bilateral hilar lymphadenopathy), CT (computed tomography), F (female), LYS (lysozyme), M (male), PPD (tuberculin test), SCAD (scudding score on chest x-ray)

All fourteen patients fulfilled the SUN classification criteria for ocular sarcoidosis.(Tables 2 and 3). The most common ocular presentations were anterior uveitis (n = 7, 50%), and panuveitis (n = 4, 28.6%). Uveitis was granulomatous in 4 (28.6%) patients. Anterior chamber cells were detected in 12 patients (85.6%). Adnexal manifestations included dacryoadenitis (n = 2, 14.3%), and conjunctival granuloma (n = 1, 7.1%). Only two patients (14.3%) presented clinically relevant keratoconjunctivitis sicca. Six (42.8%) of the biopsy confirmed patients debuted with ocular manifestations, all developed systemic manifestations of sarcoidosis during follow-up. (Table 3).

**Table 3:** Patient ocular and systemic manifestations

Ocular signs	n patients (%)	Systemic manifestations	n patients (%)
Anterior uveitis	7 (50%)	**Pulmonary**	7 (50%)
Mutton fat keratic precipitates	4 (28.6%)	-Dyspnea	7 (50%)
Fine keratic precipitates	7 (50%)	-Chest Pain	6 (42.8%)
Koeppe nodules	3 (21.4%)	-Cough	7 (50%)
Bussaca nodules	1 (7.1%)	**General**	8 (57.1%)
Berlin nodules	1 (7.1%)	-Fever	3 (21.4%)
Intermediate uveitis	1 (7.1%)	-Fatigue	8 (57.1%)
Vitreous haze	5 (35.7% )	-Weight loss	4 (28.6%)
Snowballs	3 (21.4%)	**Skin**	9 (64.3%)
Snowbanks	1 (7.1%)	-Granulomas	7 (50%)
Posterior uveitis	2 (14.3%%)	-Erythema Nodosum	4 (28.6%)
Chorioretinal lesions	5 (14.3%)	-Plaques	3 (21.4%)
Papillitis	2 (14.3%)	-Annular lesions	4 (28.6%)
Candle wax lesions	2 (14.3%)	**Neurologic**	2 (14.3%)
Periphlebitis	4 (28.6%)	-VII nerve palsy	1 (7.14%)
Panuveitis	4 (28.6%)	-Paresthesia	1 (7.14)
Imaging signs	n patients (%)	**Muscle-Skeletal**	8 (57.1%)
Apical changes	2 (14.3%)	-Arthralgia	8 (57.1%)
Volume reduction	6 (42.8% )	**Endocrine**	3 (21.4%)
Peribronchial thickening	3 (21.4%)	-Parotiditis	3 (21.4%)
Interstitial infiltrates	7 (50%)	**Syndromes**	2 (21.4%)
Atelectasis	6 (42.8%)	-Löfgren	1 (7.1%)
Perilymphatic nodules	6 (42.8% )	-Heerfordt	1 (7.1%)
Micronodules	6 (42.8%)		
BHL	9 (64.3%)		

*BHL (Bilateral Hilar Lymphadenopathy)

All patients had systemic involvement; most commonly general symptoms (n = 8, 57.1%), and pulmonary manifestations (n = 7, 50%) (Tables 2 and 3).

ACE serum levels were available in 10 (71.1%) patients, 6 (60%) had increased levels, with a median concentration of 70.5 (38.6; 89) UI/l. Lysozyme was available in 8 patients (57.1%), showing high levels in 5 cases (62.5%) with a median of 3.81 (5.6; 24) mg/L. PPD was available for 13 patients; one had a positive result, so tuberculosis was discarded by clinical picture, negative IGRA, and a positive skin lesion biopsy for sarcoidosis [2]. Patient biopsies were collected from the skin (n = 5, 55%), lacrimal glands (n = 2, 22%), bronchi (n = 2, 22%), and vitreous body (n = 1, 11%).

A chest X-ray was obtained in 9 patients (64.3%) and a CT scan for 13 patients (92.8%). Both studies were available in 8 patients. (Table 3)

All patients received treatment. (Table 4) Topical 1% prednisolone (64.28%) and oral prednisone (92.8%) were the most common initial treatment. Immunomodulators were prescribed to 12 (85.7%) patients, including azathioprine (n = 6, 42.8%), methotrexate (n = 6, 42.8%), and adalimumab (n = 1, 7.1%); these allowed early removal of corticosteroid therapy in 6 patients (42.8%) with adequate response. These medications were started due to ocular manifestations in eight (57.1%) patients. On their last visit, 10 (71.42%) patients had remission of both systemic and ocular inflammation, 4 (28.6%) patients remained with ocular inflammation, and one as worsening activity of uveitis. Treatment for patients with persistent inflammation consisted of prednisone (n = 4, 100%), azathioprine (n = 2, 50%), methotrexate (n = 3, 75%), and topical steroids (n = 2, 50%).

**Table 4:** Patients treatment, visual outcome, and ocular complications

Patient	Age	Sex	Treatment	Initial BCVA	Final BCVA	Outcome*, complication
1	53	F	PDN, PRDL, MTX, AZT	20/20 20/20	20/20 20/20	Inactive disease, cataract
2	70	F	PDN, PRDL	20/25 20/20	20/40 20/30	Inactive disease, no complications
3	28	M	PDN, IVMP (3), AZT, PRDL	20/40 20/70	20/20 20/60	Inactive disease, cataract, glaucoma
4	70	F	PDN, MTX, HCQ, PRDL	20/50 20/30	20/20 20/40	Inactive disease, posterior synechiae
5	56	F	PDN, MTX, PRDL	20/30 20/150	20/25 20/25	Inactive disease, cataract
6	66	F	AZT, PDN, PRDL	20/70 20/50	20/60 20/50	Inactive disease, no complications
7	42	F	PDN	20/30 20/30	20/20 20/20	Inactive disease, cataract
8	21	M	PDN, AZT	20/20 20/20	20/20 20/20	Improved activity, posterior synechiae
9	24	M	PDN, MMF, ADA	HM HM	HM HM	Inactive disease, glaucoma, corneal leukoma
10	68	F	MTX, PRDL	20/70 20/30	20/80 20/80	Inactive disease, glaucoma
11	67	F	PDN, MTX, PRDL	20/60 20/200	20/30 20/40	Improved activity, glaucoma
12	35	M	PDN, AZT, PRDL	20/20 20/25	20/30 20/30	Improved activity, cataract
13	47	F	PDN, AZT	20/30 20/30	20/80 20/80	Inactive disease, no complications
14	51	F	PDN, MTX	20/50 20/50	20/200 20/200	Worsening activity, cataract

ADA (adalimumab), AZA (azathioprine), HCQ (hydroxychloroquine), IVMP (intravenous methylprednisolone), MMF (mycophenolate mofetil), MTX (methotrexate), PDN (prednisone), PRDL (topical prednisolone acetate)

Nine (64.28%) patients with a definite diagnosis had long-term ocular complications (Table 4). At the end of the follow-up, eleven eyes (39.28%) had a visual outcome worse than 20/50, and seventeen eyes (60.7%) had an outcome better than 20/50 (logMAR 0.39).

## Discussion

This study describes the clinical characteristics of 14 patients diagnosed with sarcoidosis. This is the largest series of Mexican subjects with ocular sarcoidosis to date, as previous Mexican studies have only reported 5 cases of patients with ocular disease [4, 8–14]. Our institution is a reference center for ocular inflammatory diseases in our country, which allows us to analyze some of the less common diseases in our population, such as sarcoidosis.

In this sample of patients, we found a greater proportion of female patients with either definite (70%) or presumed (75%) sarcoidosis, which is similar to previous international reports (Table 5). Our patients’ most common extraocular manifestations are similar to the previous Mexican case series of systemic sarcoidosis [4].

**Table 5:** Ocular Sarcoidosis around the world

Characteristic		International study (Acharya, et al.) (20)	South India (Kalpana, et al.)(27)	Germany (zur Bonsen) (30)	France (Rochepeau et al.) (19)	France (Coulon et al.) (17)	Mexico (APEC)
n		180 (167*)	61	84	83	194	14
Age		49 (39–60)	43 (± 16.5)	53 (8–87)	52 (37–62)	52	52 (34; 67)
Female		115 (68.8%)	37(60.7%)	50 (59.5%)	64 (77.1%)	134 (69%)	10 (71.4%)
High ACE (UI/L)		80 (47.9%)	34 (59.6%)	32 (38.4%)	53 (67.1%)	113 (58%)	5 (35.7%)
High LYS (UI/L)			-	-	53 (88.3%)	112 (57%)	5 (35.7%)
Leading ocular manifestation		167 (100%)	-	53 (63%)	62 (75%)	194 (100%)	10 (71.4%)
Bilateral		142 (85%)	42 (68.9%)	62 (73.8%)	63 (75.9%)	151 (77.8%)	13(92.8%)
Löfgren syndrome		-	-	-	-	-	1 (7.1%)
Heerfordt syndrome		-	-	-	-	-	1(7.1%)
	Ocular outcome						
< 20/200		-	-	-	2 (2.4%)	21 (11%)	2 (14.3%)
< 20/50		-	-	-	9 (10.8%)	64 (33%)	4 (28.6%)
> 20/50		-	-	-	50 (60.2%)	109 (56%)	8 (57.1%)
Uveitis			61 (100%)	84 (100%)	83 (100%)	194 (100%)	14 (100%)
Anterior		34 (20.3%)	19 (31.1%)	26 (31%)	28 (33.7%)	66 (34%)	7 (50%)
Intermediate		5 (2.9%)	7 (11.5%)	27 (32.1%)	4 (4.8%)	20 (10%)	2 (14.3.%)
Posterior		15 (8.9%)	2 (3.3%)	14 (17.6%)	11 (13.2%)	14 (7.5%)	1 (7.1%)
Panuveitis		113 (67.6%)	33 (54.1%)	16 (19%)	40 (48.1%)	94 (48.5%)	4 (28.6%)
	IWOS						
Probable		13 (7.2%)	-	27 (32.1%)	-	-	-
Presumed		69 (38.3%)	-	33 (39.3%)	-	49 (25.3%)	4 (28.6%)
Definite		98 (54.4%)	61 (100%)	24 (28.6%)	83 (100%)	145 (74.7%)	10 (71.4%)

– not reported. * Patients analyzed ACE (angiotensin converting enzyme), LYS (lysozyme)

Anterior uveitis was the most common anatomical location, which contrasts with previous international reports of ocular sarcoidosis [19, 20, 29, 30]. (Table 5). Even though keratoconjunctivitis sicca is one of the most common classical presentations in sarcoidosis, only two patients had clinically relevant findings supporting this condition.

All our patients had systemic involvement and activity of the disease compared to previous reports with ranges from 36.6 to 62.6% [17, 19] probably due to the sample size and that our patients had ocular manifestations that led them to seek medical attention. The main clinical signs of systemic involvement were like international reports, involving the skin, musculoskeletal and pulmonary [17, 19].

There are plenty of studies that show an ample group of ocular manifestations as the leading manifestation to diagnose sarcoidosis, ranging from 63 to 100% [19, 20, 30]. Our patients are within that range, finding that 71.4% of patients had ocular activity as the first detectable change in sarcoidosis [31, 32]. For this reason, ophthalmologists should pay attention to early signs of ocular sarcoidosis, as prompt treatment may avoid systemic activity.

Other aspects of patient history, such as tattoos, are associated with cutaneous sarcoidosis, systemic sarcoidosis with uveitis, and isolated sarcoid uveitis [33]. In our study, two patients had permanent tattoos and did not have a reactivation or disease worsening during the follow-up.

The diagnosis of sarcoidosis is often confused with tuberculosis in endemic countries, [27] and Mexico is among the top five countries with the most cases of tuberculosis in America [34]. There is a diagnostic challenge differentiating the uveitic picture caused by both diseases, and an erroneous diagnosis of tuberculosis could be the reason for the low prevalence of sarcoidosis in Mexico.

We can observe in this study that ocular sarcoidosis can precede systemic manifestations. We must consider sarcoidosis as a differential diagnosis in patients with inflammatory eye diseases, start the diagnostic approach as an ophthalmologist and rely on a multidisciplinary group for proper systemic diagnosis and treatment.

Some of the strengths of the present study are that this is the largest group of sarcoidosis reported in the Mexican population, which can help us identify the disease characteristics, in a tertiary ophthalmology clinic. Furthermore, almost every clinical presentation of sarcoidosis was present in our series; including classic syndromes like Löfgren and Heerfordt, which are rarely mentioned even in international studies (Table 5) [2, 21].

## Conclusions

Ocular manifestations, although diverse, can be crucial in the suspicion and diagnosis of systemic sarcoidosis. In our experience, every patient that starts as an isolated form of ocular inflammation later resulted in systemic sarcoidosis. Therefore, ophthalmologists should be attentive to the manifestations of ocular sarcoidosis, and screen for systemic involvement, along with a multidisciplinary team, when suspicion is high.

## Data Availability

The data that support the findings of this study are available from the corresponding author, LECR, upon reasonable request.

## Abbreviations

IWOS: International Criteria for the Diagnosis of Ocular Sarcoidosis
SUN: Standardization of Uveitis Nomenclature
WASOG: World Association of Sarcoidosis and Other Granulomatous Disorders
ACE: Angiotensin Converting Enzyme
LYS: Lysozyme
VDRL: Venereal disease research laboratory
PPD: Tuberculin test
BHL: Bilateral Hilar Lymphadenopathy
SCAD: Scadding score on chest X-ray
CT: Computed Tomography
PDN: Prednisone
MTX: Methotrexate
AZA: Azathioprine
HCQ: Hydroxicloroquine
MMF: Mycophenolate mofetil
ADA: Adalimumab
Bx: Biopsy
Txt: Treatment

## References

[1] Judson MA, Costabel U, Drent M, Wells A, Maier L, Koth L, et al. The WASOG Sarcoidosis Organ Assessment Instrument: an update of a previous clinical tool. Volume 13. Sarcoidosis Vasculitis And Diffuse Lung Diseases; 2014. 24751450

[2] Jabs D, Acharya N, Denniston A, Lightman S, McCluskey P, Oden N et al. Classification Criteria for Sarcoidosis-Associated Uveitis. Am J Ophthalmol 2021 Aug 1;228:220–30. 10.1016/j.ajo.2021.03.047 PMC8594768 33845001

[3] Uveitis: Autoimmunity… and beyond. Autoimmun Rev. 2019 Sep 1;18(9):102351. 10.1016/j.autrev.2019.102351 31323361

[4] Carrillo-Pérez DL, Apodaca-Cháveza EI, Carrillo-Maravilla E, Mejía-Ávila M, Hernández-Oropeza JL, Reyes E (2015). Sarcoidosis: a single hospital-based study in a 24-year period. Rev Invest Clin.

[5] Jiménez-Balderas FJ, Fernandez-Arrieta G, Camargo-Coronel A, Ake-Uc M, Vazquez-Zaragoza M, Zonana-Nacach A, et al. Uveitis in Adult Patients with Rheumatic Inflammatory Autoimmune Diseases at a tertiary-care hospital in Mexico City. J Rheumatol. 2011 Feb;38(2):325–30. 10.3899/jrheum.100015 21123327

[6] Cozier YC. Assessing the worldwide epidemiology of sarcoidosis: challenges and future directions. Eur Respir J 2016 Dec 1;48(6):1545–8. 10.1183/13993003.01819-2016 27903684

[7] Arkema EV, Cozier YC. Sarcoidosis epidemiology: recent estimates of incidence, prevalence and risk factors. Curr Opin Pulm Med. 2020 Sep;26(5):527–34. 10.1097/MCP.0000000000000715 PMC7755458 32701677

[8] Arévalo-López A, Macotela-Ruiz E (1991). [Sarcoidosis (intra- and extrathoracic): apropos a mexican case]. Gac Med Mex.

[9] Estrada-Correa G, Molina-Carrión LE, Ysita-Morales A (2006). [Neurosarcoidosis. Report of one case in Mexico]. Rev Med Inst Mex Seguro Soc.

[10] Fernández-Sánchez M, Saeb-Lima M. Sarcoidosis cutánea Informe de un caso. Volume 50. Rev Med Inst Mex Seguro Soc; 2012. 23282269

[11] Peralta-Gómez MY, Ávila-Ocampo KA, Huerta-Velázquez S, Rivera-Salgado MI. Sarcoidosis de la glándula lagrimal como primera manifestación. Arch Soc Esp Oftalmol. 2018 Apr 1;93(4):206–8. 10.1016/j.oftal.2017.05.001 28610808

[12] Vega-Cornejo G, Ayala-Buenrostro P. Laryngeal sarcoidosis in a child: case report. Reumatol Clínica (English Ed. 2019 Nov;15(6):e102–4. 10.1016/j.reuma.2017.08.014 29102585

[13] Almaguer-Morales D, Hinojosa-González DE, Garza-Alpirez A. Löfgren Syndrome with Hypercalcemia and Neuroendocrinological involvement: a Case Report. Curr Rheumatol Rev 2020 Dec 24;16(4):337–42. 10.2174/1573397115666191021154628 31642787

[14] Solis JG, Olascoaga Lugo A, Rodríguez Florido MA, Sandoval Bonilla BA, Malagón Rangel J. Neurosarcoidosis Presentation as Adipsic Diabetes Insipidus secondary to a Pituitary Stalk Lesion and Association with Anti-NMDA receptor antibodies. Case Rep Neurol Med. 2020 Jun;25:2020:1–5. 10.1155/2020/7956350 PMC7334767 32670647

[15] Bargagli E, Prasse A. Sarcoidosis: a review for the internist. Intern Emerg Med. 2018 Jan;3(3):325–31. 10.1007/s11739-017-1778-6 29299831

[16] Jameson A, Revels J, Wang LL, Wang DT, Wang SS. Sarcoidosis, the master mimicker. Curr Probl Diagn Radiol. 2022 Jan;51(1):60–72. 10.1067/j.cpradiol.2020.10.013 33308891

[17] Coulon C, Kodjikian L, Rochepeau C, Perard L, Jardel S, Burillon C, et al. Ethnicity and association with ocular, systemic manifestations and prognosis in 194 patients with sarcoid uveitis. Graefe’s Arch Clin Exp Ophthalmol. 2019 Nov;13(11):2495–503. 10.1007/s00417-019-04415-x 31302765

[18] Sève P, Jamilloux Y, Tilikete C, Gerfaud-Valentin M, Kodjikian L. El Jammal T. Ocular Sarcoidosis. Semin Respir Crit Care Med. 2020 Oct;10(5):673–88. 10.1055/s-0040-1710536 32777852

[19] Rochepeau C, Jamilloux Y, Kerever S, Febvay C, Perard L, Broussolle C, et al. Long-term visual and systemic prognoses of 83 cases of biopsy-proven sarcoid uveitis. Br J Ophthalmol. 2017 Jul;101(1):856–61. 10.1136/bjophthalmol-2016-309767 27888183

[20] Acharya NR, Browne EN, Rao N, Mochizuki M. Distinguishing Features of Ocular Sarcoidosis in an International Cohort of Uveitis Patients. Ophthalmology. 2018 Jan 1;125(1):119–26. 10.1016/j.ophtha.2017.07.006 28823384

[21] Mochizuki M, Smith JR, Takase H, Kaburaki T, Acharya NR, Rao NA. Revised criteria of International Workshop on Ocular Sarcoidosis (IWOS) for the diagnosis of ocular sarcoidosis. Br J Ophthalmol. 2019;103(10):1418–22. 10.1136/bjophthalmol-2018-313356 30798264

[22] Sève P, Pacheco Y, Durupt F, Jamilloux Y, Gerfaud-Valentin M, Isaac S et al. Sarcoidosis: a clinical overview from symptoms to diagnosis. Cells. 2021;10(4). 10.3390/cells10040766 PMC8066110 33807303

[23] Takase H. Characteristics and management of ocular sarcoidosis. Immunol Med. 2022 Jan;2(1):12–21. 10.1080/25785826.2021.1940740 34214013

[24] Jabs DA, Nussenblatt RB, Rosenbaum JT, Atmaca LS, Becker MD, Brezin AP et al. Standardization of Uveitis Nomenclature for Reporting Clinical Data. Results of the First International Workshop. Am J Ophthalmol. 2005 Sep 1;140(3):509–16. 10.1016/j.ajo.2005.03.057 PMC8935739 16196117

[25] Crouser ED, Maier LA, Baughman RP, Abston E, Bernstein RC, Blankstein R, et al. Diagnosis and detection of sarcoidosis an official american thoracic Society Clinical Practice Guideline. American Journal of Respiratory and Critical Care Medicine. Volume 201. American Thoracic Society; 2020. pp. E26–51. 10.1164/rccm.202002-0251ST PMC7159433 32293205

[26] Thillai M, Atkins CP, Crawshaw A, Hart SP, Ho LP, Kouranos V et al. BTS Clinical Statement on pulmonary sarcoidosis. Thorax. 2021 Jan 1;76(1):4–20. 10.1136/thoraxjnl-2019-214348 33268456

[27] Babu K, Biswas J, Agarwal M, Mahendradas P, Bansal R, Rathinam SR et al. Diagnostic markers in Ocular Sarcoidosis in A High TB Endemic Population – A Multicentre Study. Ocul Immunol Inflamm 2022 Jan 2;30(1):163–7. 10.1080/09273948.2020.1778733 32870050

[28] Drent M, Vries J, De, Lenters M, Lamers RJS, Rothkranz-Kos S, Wouters EFM, et al. Sarcoidosis: assessment of disease severity using HRCT. Eur Radiol. 2003 Nov;17(11):2462–71. 10.1007/s00330-003-1965-x 12811502

[29] Clement DS, Postma G, Rothova A, Grutters JC, Prokop M, de Jong PA. Intraocular sarcoidosis: association of clinical characteristics of uveitis with positive chest high-resolution computed tomography findings. Br J Ophthalmol. 2010 Feb;94(1):219–22. 10.1136/bjo.2009.161588 19713200

[30] zur Bonsen LS, Pohlmann D, Rübsam A, Pleyer U. Findings and graduation of sarcoidosis-related Uveitis: a single-center study. Cells. 2021 Dec 29;11(1):89. 10.3390/cells11010089 PMC8750073 35011651

[31] Collison JMT, Miller NR, Green WR (1986). Involvement of Orbital Tissues by Sarcoid. Am J Ophthalmol.

[32] Ma SP, Rogers SL, Hall AJ, Hodgson L, Brennan J, Stawell RJ, et al. Sarcoidosis-related Uveitis: clinical presentation, Disease Course, and rates of systemic Disease Progression after Uveitis diagnosis. Am J Ophthalmol. 2019 Feb;198:30–6. 10.1016/j.ajo.2018.09.013 30243930

[33] Kluger N. Tattoo-associated uveitis with or without systemic sarcoidosis: a comparative review of the literature. J Eur Acad Dermatology Venereol 2018 Nov 29;32(11):1852–61. 10.1111/jdv.15070 29763518

[34] Zenteno-Cuevas R, Munro-Rojas D, Pérez-Martínez D, Fernandez-Morales E, Jimenez-Ruano AC, Montero H et al. Genetic diversity and drug susceptibility of Mycobacterium tuberculosis in a city with a high prevalence of drug resistant tuberculosis from Southeast of Mexico. BMC Infect Dis. 2021 Dec 30;21(1):1202. 10.1186/s12879-021-06904-z PMC8630842 34847856

